# Acuity and Quality of Vision in Eyes with Epithelial Basement Membrane Dystrophy after Regular Pseudophakia

**DOI:** 10.3390/jcm12031099

**Published:** 2023-01-31

**Authors:** Carlo Bellucci, Paolo Mora, Salvatore A. Tedesco, Arturo Carta, Stefano Gandolfi, Roberto Bellucci

**Affiliations:** 1 Ophthalmology Unit, University Hospital of Parma, 43126 Parma, Italy; 2 Vista Vision Surgical Centre, 37135 Verona, Italy

**Keywords:** corneal dystrophies, pseudophakia, visual impairment, aberrometry

## Abstract

Purpose: This retrospective case-control study was conducted to quantitatively and qualitatively assess the visual impairment in eyes with Epithelial Basement Membrane Dystrophy (EBMD) after regular cataract surgery. Methods: EBMD pseudophakic eyes were compared with matched pseudophakic eyes free from surface disorders. At least 3 weeks after surgery we evaluated uncorrected and best-corrected distance visual acuity (UDVA and CDVA), objective aberrometry, Point Spread Function (PSF), Modulation Transfer Function (MTF), and patient complaints. Results: Twenty-five EBMD eyes and 25 control eyes (13 patients per group) were included. Nine patients per group had a monofocal IOL, and four patients had a trifocal IOL. All the EBMD patients complained of postoperative blurred vision with ocular discomfort; intensive use of lubricants induced subjective improvement only in eyes with monofocal IOLs. Postoperative mean UDVA was 0.19 ± 0.16 LogMAR in the EBMD eyes and 0.11 ± 0.04 LogMAR in the control group (*p* = 0.016). Mean CDVA was 0.18 ± 0.15 LogMAR in the EBMD eyes and 0.06 ± 0.04 LogMAR in the control eyes (*p* = 0.001). The PSF curve width was significantly worse in the EBMD group (*p* < 0.001). The MTF cut-off value was lower in the EBMD group than in the control group (*p* < 0.001). Conclusion: After cataract removal, eyes with EBMD had significantly lower UDVA and CDVA than controls. All the aberrometric parameters were significantly worse in EBMD cases. EBMD patients complained about their postoperative visual outcome, while control patients did not.

## 1. Introduction

Epithelial Basement Membrane Dystrophy (EBMD), also termed Map-Dot fingerprint dystrophy and Cogan’s Dystrophy, is a corneal surface disorder mainly involving adulthood. Affected patients usually report mild visual blurring or foreign body sensations, or occasional pain immediately upon the opening of the lids at night or in the morning [1]. The typical slit-lamp appearance consists of irregular, amorphous, clear zones on the corneal surface, sharply demarcated within light greyish areas (“maps”); small irregular putty-like greyish/white opacities (“dots”); and small clusters of curved parallel lines (“fingerprint”) [2].

The disease can improve with lubricants, but the actual resolution does not occur. In cases presenting recurrent corneal erosions, laser phototherapeutic keratectomy (PTK) is recommended [3].

Because the symptoms overlap with those of dry eye and the variable evidence, except for some cases of the familiar pattern, EBMD is often ignored by affected patients and/or overlooked by general ophthalmologists [4]. Studies about the visual impairment of EBMD are progressively increasing but none have addressed the functional impact of EBMD specifically on pseudophakic vision yet. In the present era of “refractive” cataract surgery, we think particularly anterior segment specialists may wish to gather data from our experience on a group of pseudophakic eyes with EBMD that have undergone an objective optical quality evaluation.

## 2. Materials and Methods

This retrospective, case-control study adhered to the declaration of Helsinki and was approved by the local Ethical Committee (#126/2022).

**Figure 1:**
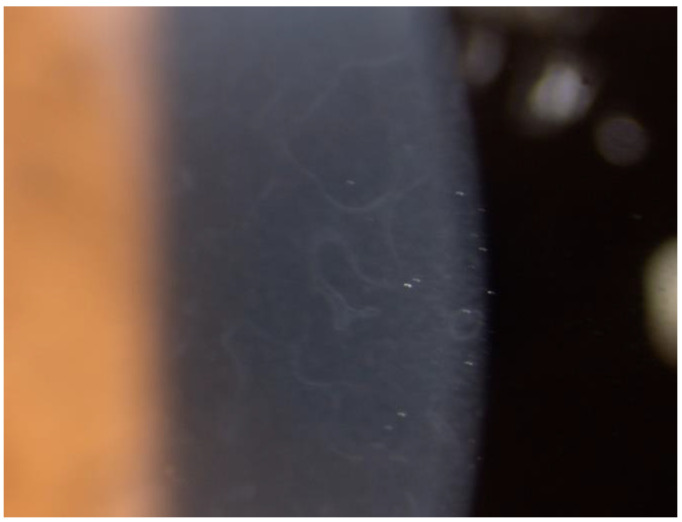
Anterior segment color photograph of Epithelial Basement Membrane Dystrophy one of the eyes included in this study.

**Figure 2:**
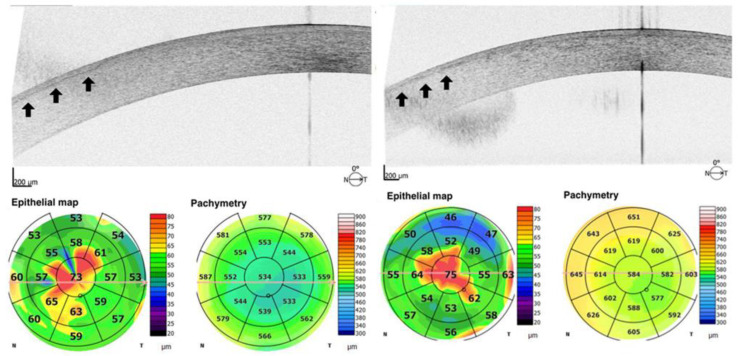
Anterior segment optical coherence tomography (AS-OCT) scans of irregular and thickened epithelial basement membrane in eyes with corneal Epithelial Basement Membrane Dystrophy (arrows). Epithelial map and pachymetry of the corresponding eye are shown at the bottom.

We selected patients who were followed for EBMD and who had undergone uneventful cataract surgery. The included patients had not been diagnosed having EBMD before cataract surgery, so they can be considered as silent cases when phakic. They had been operated on by two surgeons (RB and SAT) who made the EBMD diagnosis at the time of the postoperative follow up. Anterior segment color photographs (Figure 1) and anterior segment optical coherence tomography (AS-OCT) scans (Figure 2) had to be available to confirm EBMD diagnosis and to allow for the evaluation of single-case details. For the study, we considered the uncorrected- and best-corrected distance visual acuity (UDVA and CDVA) and the aberrometry performed at least 3 weeks after the phacoemulsification in the affected eye. Optical quality was evaluated, by means of the Optical Quality Analysing System (OQAS II, HD Analyzer, Visiometrics SL, Terrassa, Spain). It is a double-pass aberrometer, i.e., it records and analyses images of a monochromatic point source after reflection on the retina and a double pass through the ocular media, which allows for measurement of the combined effects of higher order aberrations and light diffusion phenomena [5,6]. The following parameters, conventionally addressed in our acquisition protocols, were collected for the included eyes with a pupil diaphragm at 4 mm:

Objective Scattering Index (OSI): assesses the light scattering produced by the optical system of the examined eye; the lower the better.

Point Spread Function (PSF 50% and 10%): considers the width of the PSF curve (arc min) at 50% and 10% of its height, respectively; the lower the better.

Modulation Transfer Function (MTF) cut-off: considers the spatial frequency (cycles/degree) at which the MTF curve falls to zero; the higher the better.

VA 100, 20, 9: refers to the potential visual acuity at 100%, 20%, and 9% contrast; the higher the better.

As the control group, an equal number of pseudophakic eyes free from any surface pathology were selected among those already present in the aberrometer database with similar age, intraocular lens (IOL) type and power.

Means and standard deviations were reported for continuous variables with a normal distribution (Shapiro–Wilk test). To analyze the data of the cases and matched control eyes, the Student’s *t*-test was used. Statistical analyses were performed using commercial software (SPSS version 25.0; IBM, Armonk, NY, USA); the *p* < 0.05 was considered significant.

## 3. Results

Twenty-five eyes from 13 EBMD patients (five men and eight women, mean age 69 years within the range 52–80 years) met the inclusion criteria and formed the study cases. Data of an equal number of eyes from 13 subjects (7 men and 6 women, mean age 72 years within the range 56–85 years) were selected from the OQAS database based on IOL type and power matching to form the control cohort. In these subjects, EBMD and severe dry eye were excluded. The case group had received a monofocal implant in 17 eyes of 9 patients, and a trifocal IOL in eight eyes of four patients (same distribution in the control group) with a mean (±SD) power of 20.6 ± 3.6 D (21.3 ± 2.5 D in the control group, *p* > 0.5). Among clinical notes, all the EBMD patients requested unscheduled postoperative visits complaining of blurred or fluctuating vision and ocular discomfort. The daytime use of lubricant tears and ointments at night was prescribed in all cases. After 3 months of this regimen, there was a related subjective improvement of visual disturbances in eyes implanted with a monofocal IOL, including one patient with a unilateral implant. Instead, no amelioration of the blurred vision and reading ability was reported by patients implanted with trifocal IOLs. All the EBMD eyes except one underwent a laser capsulotomy within 6 months after phacoemulsification without functional and/or subjective symptoms improvement.

**Figure 3:**
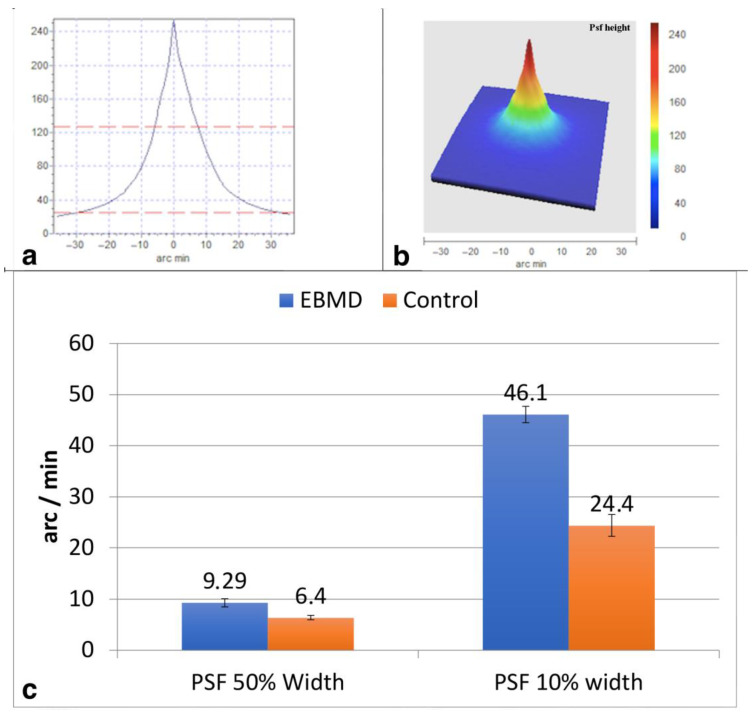
Bidimensional (**a**), tridimensional (**b**) and values (**c**) of the mean Point Spread Function (PSF) for Epithelial Basement Membrane Dystrophy (EBMD) eyes. At 50%, the width of the PSF curve of the EBMD eyes was 1.5 times larger than that of control eyes. At 10%, the width of the PSF curve of the EBMD eyes was two times larger than that of control eyes.

**Figure 4:**
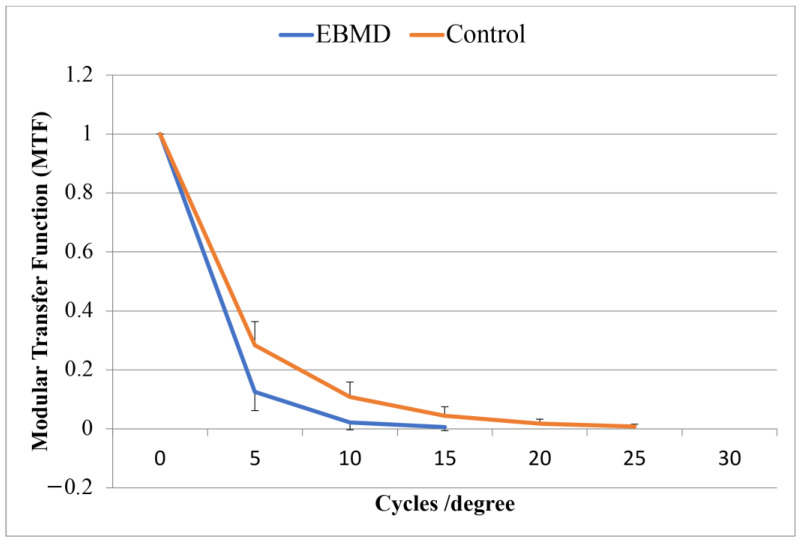
The Modular Transfer Function (MTF) cut-off for the two groups was averagely lower in the case group than in the control group.

The mean UDVA was 0.19 ± 0.16 LogMAR in the EBMD eyes and 0.11 ± 0.04 LogMAR in the control group (*p* = 0.016). The mean CDVA was 0.18 ± 0.15 LogMAR in the EBMD eyes and 0.06 ± 0.04 LogMAR in the control eyes (*p* = 0.001). The double-pass aberrometer evaluation provided an OSI at 4.38 ± 0.80 in the EBMD eyes, and at 2.12 ± 0.44 in the control eyes (*p* < 0.001). The width of the PSF curve of the EBMD eyes (Figure 3) was 1.5 times and 2 times larger than that of the control eyes at 50% and at 10% of the curve height, respectively (*p* < 0.001). The MTF curves (mean values at tested spatial frequencies) in the two groups are shown in Figure 4. The MTF cutoff was at 13.7 ± 4.2 cycles/degree in the EBMD eyes and at 29.2 ± 6.0 cycles/degree in the control eyes (*p* < 0.001).

**Figure 5:**
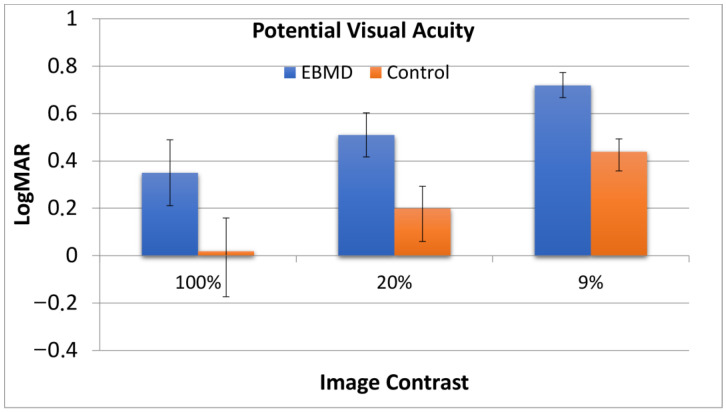
Potential visual acuity for EBMD patients and control group at 100%, 20%, and 9% image contrast, computed from the MTF function. All the differences between the groups were statistically significant with the highest difference recorded for 100% image contrast.

The potential visual acuity as computed by the machine from the MTF function is reported in Figure 5 for 100%, 20%, and 9% image contrast. All the differences between the groups were statistically significant at the *p* < 0.001 level; the highest difference was recorded for 100% image contrast. The small number of cases made the intra-group statistical comparison between monofocal and multifocal IOL cases poorly reliable.

## 4. Discussion

This retrospective, case-control study addressed the visual acuity and quality results in EBMD eyes after regular phacoemulsification for senile cataracts in a single-centre practice.

Based on recent literature, EBMD was recorded in 7.5% of patients presenting ocular surface dysfunction in a Northern American cohort of subjects requesting cataract surgery evaluation [7]. Although the prevalence of the disorder in the population of our country is not specifically stated, it is reasonable to expect that a non-negligible proportion of patients referred for cataract surgery may be affected by EBMD. The accuracy of measurements obtainable with new technological tools such as AS-OCT and OQAS has certainly stimulated recent interesting publications on the topic [8]. To our knowledge, this is the first evaluation of the objective quality of vision in pseudophakic EBMD eyes.

From our clinical and instrumental findings, the following observations can be put forward. There is a consistent probability that EBMD patients will obtain a significantly lower UDVA and, even more, CDVA than eyes without this surface alteration. This is not fully attributable to errors in the preoperative calculation of the implant power, which should be of maximum care, but are from surface disturbances affecting refraction [9,10,11,12]. EBMD patients on average will require unscheduled postoperative visits and an early capsulotomy in the absence of known surgery complications. Such evidence should bring attention to this possible diagnosis if it is not already known at the time of surgery. An intensive course of lubricant supplementation can ameliorate postoperative symptoms, chiefly in eyes receiving monofocal IOLs. The use of diffractive multifocal IOLs should be carefully considered and rather limited in these patients [13]. All the considered aberrometric parameters tested significantly worse in EBMD cases than in the control eyes. The visual performance related to the contrast of the images, upon testing, was particularly concerning. It is conceivable that surgery may affect both the refractive outcome and corneal transparency, especially in case of multifocal IOLs hence leading to non optimal visual performance patient complaints [11].

Among the study limitations, there are the retrospective design and the limited sample size. This prevented a further comparison between cases by addressing those implanted with monofocal vs. those with multifocal IOLs. In addition, the presence of bilateral cases is generally poorly desirable for statistics; however, the relative rarity of the form and the fact that subjective functional outcomes are best judged after bilateral implantation (especially for multifocal IOLs), led us to include these cases.

EBMD is frequently misdiagnosed in the elderly, and even at the time of the pre-operative work-up before cataract surgery. The number of silent cases probably overcomes the number of symptomatic cases, and the symptoms may develop months or years after surgery. When EBMD is diagnosed in a pseudophakic patient, the suspect arises that cataract surgery favored the development of the disorder.

In conclusion, high importance should be given to the screening for EBMD when assessing a patient for cataract surgery, which is mandatory when we select a patient for multifocal lens implantation. When our patient asks for these IOLs and an EBMD is found, we must be very clear with him immediately and take our time to explain why his eye, to his knowledge healthy apart from the cataract, may not be suitable for the multifocal implantation.

In such conditions, treatment with lubricants, hypertonic saline solution, or application of surface ablation procedures in anticipation of multifocal lens implantation may be a useful strategy, although considering that there is currently no universal guideline for the surgical management of patients with EBMD. Dialogue with the patient in order to clarify the diagnosis remains the key point to avoid unexpected and unpleasant outcomes for both the ophthalmologist and the patient.

## Data Availability

All data and material are available from the corresponding author.
